# High drug payload curcumin nanosuspensions stabilized by mPEG-DSPE and SPC: *in vitro* and *in vivo* evaluation

**DOI:** 10.1080/10717544.2016.1233589

**Published:** 2017-02-03

**Authors:** Jingyi Hong, Yingying Liu, Yao Xiao, Xiaofeng Yang, Wenjing Su, Mingzhu Zhang, Yonghong Liao, Haixue Kuang, Xiangtao Wang

**Affiliations:** 1 Key Laboratory of Bioactive Substances and Resources Utilization of Chinese Herbal Medicine, Institute of Medicinal Plant Development, Chinese Academy of Medical Sciences & Peking Union Medical College, Beijing, China,; 2 School of Pharmacy, Heilongjiang University of Chinese Medicine, Harbin, China, and; 3 Life Science and Environmental Science Center, Harbin University of Commerce, Harbin, China

**Keywords:** Curcumin, nanosuspensions, high drug payload, pharmacokinetics, biodistribution, antitumor efficacy

## Abstract

*Context*: Curcumin (CUR) is a promising drug candidate based on its broad bioactivities and good antitumor effect, but the application of CUR is potentially restricted because of its poor solubility and bioavailability.

*Objective*: This study aims at developing a simple and effective drug delivery system for CUR to enhance its solubility and bioavailability thus to improve its antitumor efficacy.

*Materials and methods:* Curcumin nanosuspensions (CUR-NSps) were prepared by precipitation-ultrasonication method using mPEG2000-DSPE and soybean lecithin as a combined stabilizer.

*Results*: CUR-NSps with a high drug payload of 67.07% were successfully prepared. The resultant CUR-NSps had a mean particle size of 186.33 ± 2.73 nm with a zeta potential of −19.00 ± 1.31 mV. *In vitro* cytotoxicity assay showed that CUR-NSps exhibited enhanced cytotoxicity compared to CUR solution. The pharmacokinetics results demonstrated that CUR-NSps exhibited a significantly greater AUC_0–24_ and prolonged MRT compared to CUR injections after intravenous administration. In the biodistribution study, CUR-NSps demonstrated enhanced biodistribution compared with CUR injections in liver, spleen, kidney, brain, and tumor. The CUR-NSps also showed improved antitumor therapeutic efficacy over the injections (70.34% versus 40.03%, *p* < 0.01).

*Conclusions*: These results suggest that CUR-NSps might represent a promising drug formulation for intravenous administration of CUR for the treatment of cancer.

## Introduction

Curcumin (CUR), a natural compound isolated from the root of *Curcuma longa* L. (Zingiberaceae family), has attracted much attention in recent years due to its surprisingly wide range of pharmacological activities, including cardio-protection, antithrombus, antioxidation, antiproliferation, antiinflammation, nephro-protection, myocardio-protection, and so on (Egan et al., 2004; Anand et al., 2007). These multiple therapeutic activities of CUR are mainly due to its potential to modulate the various signaling pathways, such as nuclear NF-κβ, P-gp, protein kinase C, ATPase, COX-2, etc. (Kunnumakkara et al., 2008). CUR was also found to have good bioactivity against many cancers, such as stomach, cervix, colorectal, and skin cancers (Cheng et al., 2001; Sharma et al., 2004; Kurd et al., 2008; Singh & Singh, 2009). It has been reported that CUR suppresses the expression of epidermal growth factor receptor (EGFR) and estrogen receptors, which are cancer-relevant growth factor (Kunnumakkara et al., 2008). The broad biological activities, good safety, and low cost have also made CUR a prospective drug (Anand et al., 2007; Aggarwal & Harikumar, 2009).

Nevertheless, the application of CUR is restricted by its poor solubility and poor oral absorption in the gastrointestinal tract. Additionally, the rapid metabolism and systemic elimination are also bottlenecks for CUR to fully play its pharmacodynamic action *in vivo* (Sharma et al., 2004; Anand et al., 2007; Peng & Qian, 2014). In order to enhance the solubility and the bioavailability of CUR, a suitable vehicle or dosage form is needed. Recently, the development in the field of nanotechnology has made excellent progresses toward enhancing the solubility and the bioavailability of lipophilic drugs (Demetzos & Pippa, 2014). Various nanomedicine-based drug delivery systems such as CUR nanoparticles (Ding et al., 2014; Guo et al., 2014; Lee et al., 2015; Mukerjee et al., 2016), polymeric micelles (Cai et al., 2015; Cao et al., 2015; Duan et al., 2015), liposomes (El-Khoury & Patra, 2016), and solid dispersions (Hu et al., 2015; Mendonca et al., 2015; Teixeira et al., 2016) have been designed. These methods successfully increased the apparent solubility and bioavailability. However, there are shortcomings with these formulations, such as low drug loading capacity (most < 20%, seldom > 50%) which results in not only high dosing requirement but also large wastage of the carrier materials. Nguyen et al. have reported CUR-bound chitosan nanoparticles with a high drug payload up to 85 wt%. However, the preparation of CUR-bound chitosan nanoparticles was far from simple and no systematic *in vivo* research was conducted to verify the *in vivo* efficacy (Nguyen et al., 2015).

Nanosuspensions (NSps), which are well-known for their high drug loading capacity, is a nanoparticle system containing almost pure drug crystal and only small amounts of surfactants or polymeric materials for stabilization (Patravale et al., 2004). When formulated into NSps, there is a great increase in the drug solubility and dissolution rate for the poorly soluble drugs. This is because when the drug particles size is reduced to nanometer size, the total effective surface area is increased allowing greater interaction with the solvent (Patel & Agrawal, 2011). The reduced particle size also allows the possibility of intravenous administration of poorly soluble drugs. Furthermore, it has been widely reported that when intravenously administered, nanosuspensions could target to tumor sites through enhanced permeability and retention effect (EPR effect) (Jain, 2005).

In this study, CUR-NSps with a high drug payload of 67.07% (w/w) were prepared by a simple precipitation-combined ultrasonication method using mPEG2000-DSPE and soybean lecithin (SPC) as a combined stabilizer. Then *in vitro* study including preparation, physicochemical characteristics, stability, and cytotoxicity assay were evaluated. The pharmacokinetics, biodistribution, and *in vivo* antitumor efficacy after i.v. administration were also studied.

## Materials and methods

### Materials

CUR (purity 95.0%) was purchased from Dalian Meilun Technology Co., Ltd. (Liaoning province, China). mPEG2000-DSPE was purchased from Laysan Bio, Inc. SPC was obtained from Guangzhou Hanfang Pharmaceutical Company Ltd. Acetonitrile (HPLC grade) was purchased from Fisher Scientific (Pittsburgh, PA); 3-(4,5-dimethylthiazol-2-yl)-2,5-diphenyltetrazolium bromide (MTT) was provided by Sigma-Aldrich (St. Louis, MO). All of the other organic solvents and chemicals were of the highest commercially available grade. The water used in the experiments was deionized. All solutions used in HPLC analysis were filtered using a 0.45-μm membrane filter.

### Animals and cell culture

Sprague–Dawley (SD) male rats (165 ± 10 g) and ICR male mice (20 ± 2 g) were purchased from Vital River Laboratory Animal Technology Co., Ltd. (Beijing, China). All of the animal experiments were performed in accordance with the Guidelines for Ethical and Regulatory for Animal Experiments as defined by the Institute of Medicinal Plant Development (IMPLAD), China. The animals were acclimated for 1 week prior to the experiments with free access to water and fodder. HCT-8 (human colon carcinoma), Hela (human cervix carcinoma), HepG2 (human hepatocellular carcinoma), 4T1 (murine mammary carcinoma), and H22 (murine hepatocarcinoma) cell lines were provided by the Cell Culture Center, Institute of Basic Medical Sciences, Chinese Academy of Medical Sciences (Beijing, China). Hela, HepG2, and 4T1 were cultured with RPMI 1640 medium (GIBCO, Carlsbad, CA), and HCT-8 was cultured with DMEM medium (GIBCO, Carlsbad, CA) containing 10% fetal calf serum (GIBCO, Carlsbad, CA), penicillin (100 U/mL), and streptomycin (100 U/mL) at 37 °C and 5% CO_2_ (SANYO, Osaka, Japan).

### High-performance liquid chromatography (HPLC) analysis of curcumin

Reverse-phase HPLC (Ultimate 3000, Thermo Fisher Scientific Inc., Sunnyvale, CA) system was utilized for *in vitro* analysis detection of CUR. A Symmetry C18 column (4.6 mm × 250 mm, 5 μm, Waters, Milford, MA) was employed for *in vitro* analysis. The mobile phase was a mixture of acetonitrile and 4% acetic acid (70:30, v/v). The flow rate was set at 1.0 mL/min; the injection volume was 20 uL; and the column temperature was 25 °C. The detection was performed by UV-vis absorption at 425 nm.

A Symmetry Shield TM RP18 chromatographic column (4.6 mm × 250 mm, 5 μm, Waters, Milford, MA) was employed to detect CUR plasma samples. The mobile phase was a mixture of acetonitrile and 0.04% trifluoroacetic acid (50:50, v/v). Other conditions were consistent with *in vitro* analysis detection method.

In order to detect the contents of CUR in tissue samples, higher sensitivity and lower detection limit were necessary, so fluorescence detector (DIONEX, λ_ex_: 442 nm and λ_em_: 475 nm) was applied. Syncronis C18 (4.6 × 150 mm, 5 μm, Thermo Scientific, Sunnyvale, CA) was employed. The mobile phase was composed of (A) acetonitrile, (B) 0.5% formic acid, and (C) tetrahydrofuran. The gradient elution for liver, kidney, and tumor was: 30% A, 50% B, and 20% C between 0 and 8 min, then changed to 45% A, 10% B, and 45% C from 8 to 11 min and maintained this ratio between 11 and 20 min. The gradient elution for heart and brain was: 30% A, 50% B, and 20% C between 0 and 8 min, then changed to 60% A, 10% B, and 30% C from 8 to 9.5 min and maintained this ratio between 9.5 and 12 min, then changed to 30% A, 50% B, and 20% C from 12 to 13 min and maintained this ratio between 13 and 18 min. The gradient elution for spleen and lung was: 30% A, 50% B, and 20% C between 0 and 8 min, then changed to 45% A, 10% B, and 45% C from 8 to 11 min and to 30% A, 50% B, and 20% C from 11 to 12 min and maintained this ratio between 12 and 20 min. The flow rate for the analysis of all the tissue samples was set at 0.1 mL/min, and the injection volume was 5 μL. Other conditions were consistent with ultraviolet detection method.

### Preparation of CUR-NSps

CUR-NSps were prepared via a precipitation-ultrasonication method with modification (Han et al., 2014). Briefly, 15 mg CUR, 3 mg mPEG2000-DSPE, and 1.5 mg SPC were co-dissolved in 2 mL acetone, then the mixed solution was injected dropwise into 11 mL of distilled water at 45 ± 2 °C with ultrasonication at 250 W for 20 min (Kun Shan Ultrasonic Instruments Co., Ltd., China), followed by evaporation of acetone at 40 °C under vacuum until no organic solvent remained. The resultant CUR-NSps were directly used for subsequent studies. To determine the drug content of CUR-NSps, samples were dissolved in 9-fold volumes of acetonitrile for the disintegration of NSps and were then passed through a 0.22-μm filter before HPLC analysis. The drug loading (DL% w/w) was calculated as follows:

(1) $$\mathrm{DL}\%=W_{\mathrm{durg}}/W\times 100\%$$

where, W_durg_ was the mass of drug in CUR-NSps and W was the total weight of freeze-dried CUR-NSps containing both drug and stabilizer.

### Size and zeta potential measurement

Mean particle size and polydispersity index (PDI) of the prepared CUR-NSps were determined by dynamic light scattering (DLS) (Zetasizer Nano ZS, Malvern Instruments, UK). Zeta potential (ZP) of the CUR-NSps was measured with the same instrument. Both the particle size and zeta potential were measured in triplicate with 12 scans each at 25 °C.

### Morphology observation by transmission electron microscopy (TEM)

The morphology of CUR-NSps was observed using a JEM-1400 electron microscope (JEOL Ltd., Tokyo, Japan). One drop of the CUR-NSps (100 μg/mL) was placed on a 300-mesh copper grid, air-dried, and negatively stained with 2% (w/v) uranyl acetate for electron microscope observation.

### Differential scanning calorimetry characterization

A differential scanning calorimeter (DSC, Q200, TA Instruments, New Castle, DE) was used to obtain DSC thermal profiles of the powder samples. Samples of approximately 5 mg were placed in standard aluminum pans, sealed with a lid and tested from 0 to 400 °C at 10 °C/min under a nitrogen atmosphere. The CUR bulk powder, blank excipients (mPEG2000-DSPE and SPC), CUR-NSps, and the physical mixture of CUR bulk powder and excipients were measured under the same condition.

### X-ray diffraction (XRD) measurements

X-ray powder diffraction (XRD) was performed on an X-ray diffractometer (DX-2700, China) with Cu-Kα radiation generated at 100 mA and 40 kV. CUR bulk powder, blank excipients (mPEG2000-DSPE and SPC), CUR-NSps, and the physical mixture of CUR bulk powder and excipients were scanned over an angular range of 3–70° of 2*θ*, with a step size of 0.02° and a count time of 3 s per step.

### The stability of CUR-NSps

#### Storage stability at 4 °C

Storage stability was studied by storing CUR-NSps at 4 °C for up to 60 days. Periodically, samples were withdrawn and analyzed for size changes and particle distribution. Each measurement was performed in triplicate.

#### Stability of CUR-NSps in various physiological solutions

CUR-NSps (1 mg/mL) were mixed (1:1, v/v) with 1.8% NaCl, 10% glucose, and 2 × PBS (pH 7.4), respectively to obtain an isotonic solution and then were incubated at 37 °C. At specific time intervals, a 1 mL sample was removed and analyzed for size changes and particle distribution. Each measurement was performed in triplicate.

#### Drug content stability of CUR-NSps in plasma

About 50 μL of CUR-NSps (1 mg/mL) and free CUR (1 mg/mL, dissolved in DMSO) were mixed with rat plasma (1:4, v/v) and incubated at 37 °C. At specific time intervals, the samples were mixed with 1 mL of ethyl acetate and vortexed vigorously for 2 min then centrifuged at 10 000 rpm for 10 min. The supernatant was collected and evaporated at 40 °C under nitrogen. The residue was reconstituted in 200 μL of acetonitrile and 20 μL was injected for HPLC analysis. The concentration determined at different time-point was compared to the original one. Each measurement was performed in triplicate.

### Hemolysis assay

Fresh rat blood was centrifuged at 5000 rpm for 10 min to remove the supernatant and was successively rinsed with 0.9% NaCl and diluted to 4% (v/v). Different concentrations of CUR-NSps (0.25–4 mg/mL) which have been adjusted to isotonic were mixed with 0.5 mL of 4% red blood cell suspensions. The deionized water was used as a positive control and 0.9% NaCl as a negative one. The mixtures were incubated at 37 °C for 4 h and then were centrifuged at 5000 rpm for 5 min. The absorbance of the supernatants was recorded using an ELISA plate reader (Biotek, Winooski, VT) at 540 nm. The hemolysis percentage was calculated according to the following equation:

(2) $$\mathrm{Hemolysis}\mathrm{percentage}\left(\%\right)\;=\left(A_{\mathrm{sample}}-A_{\mathrm{negative}}\right)/\left(A_{\mathrm{positive}}-A_{\mathrm{negative}}\right)\times 100$$

in which, A_sample_ is the absorbance of the CUR-NSps, A_negative_ is the absorbance of the negative control, and A_positive_ is the absorbance of the positive control. All samples were analyzed in triplicate.

### *In vitro* cytotoxicity assay

The cytotoxicity of CUR-NSps against the Hela, 4T1, HCT-8, and HepG2 cell lines was measured using the MTT assay. Typically, 200 μL of cells (5.0 × 10^4^ cells/mL) were seeded in a 96-well plate and maintained overnight at 37 °C in 5% CO_2._ The cells were then incubated with CUR-NSps and free CUR solution (dissolved in DMSO, further diluted with culture medium, final concentration of DMSO ≤ 0.1%) at serial concentrations for 48 h. After that, the cells were treated with 20 μL of MTT solution (5 mg/mL in PBS) for another 4 h. Finally, the medium was removed and 200 μL of DMSO were added to each well to dissolve formazan crystals. The maximum absorbance was detected at 570 nm using an ELISA plate reader (Biotek, Winooski, VT):

(3) $$\mathrm{Cell}\mathrm{inhibition}\mathrm{rate}\left(\%\right)=\left(1-{\mathrm{OD}}_{t}/{\mathrm{OD}}_{c}\right)\times 100\%$$

where, OD_t_ is the mean OD of tested group and OD_c_ is the mean OD of control group.

The IC_50_ value for ACGs-NSps was determined using GraphPad Prism software, version 5, by the sigmoidal dose-response variable curve fitting method.

### Pharmacokinetic analysis

Twenty Sprague-Dawley rats were randomly divided into two groups. After 12-h fasting, CUR-NSps and CUR injections (3 mg CUR was dissolved in 0.8 mL of DMSO/Tween 80 [1:1, v/v] mixed solution and then diluted to 3 mL with saline before use) were administered via tail vein to each rat at a dose of 10 mg/kg. Blood samples (approximately 0.5 mL) were obtained at 5, 10, 20, 30, 60, 120, 240, 480, 720, and 1440 min from the orbital plexus after i.v. administration and centrifuged immediately at 5000 rpm for 5 min. Then 0.2 mL supernatant was collected and mixed with 1 mL of ethyl acetate. The mixture was vortexed vigorously for 2 min and centrifuged at 10 000 rpm for 10 min. The supernatant was collected and evaporated at 40 °C under nitrogen. The residue was reconstituted in 200 μL of acetonitrile, and 20 μL was injected for HPLC analysis. All parameters are calculated by using Phoenix WinNonlin software, version 6.1.

### *In vivo* biodistribution study

Five ICR mice (6–8 weeks old) were administered with 0.1 mL of H22 cells (10^7^ cells/mL) via intraperitoneal injection. After 7 days, ascites were collected, washed twice, and diluted with PBS. Subsequently, 0.2 mL of H22 cells (10^7^ cells/mL) was injected subcutaneously into 100 ICR mice in the right armpit. When the size of tumors reached 100 mm^3^, 72 H22 tumor-bearing mice with relatively uniform tumor volume were selected for the following experiment.

The H22 tumor-bearing mice were randomly divided into two groups (36 mice each) and were intravenously administrated with CUR-NSps or CUR injections (3 mg CUR was dissolved in 0.8 mL of DMSO/Tween 80 (1:1, v/v) mixed solution and then diluted to 3 mL with saline before use) through tail vein at a dose of 8.0 mg/kg. At each predetermined time intervals (0.5 h, 2 h, 4 h, 8 h, 12 h, and 24 h), 6 mice in each group were taken out and sacrificed. Then the heart, liver, spleen, lung, kidney, brain, and tumors were excised immediately, rinsed with normal saline, dried with filter paper, and homogenized with 3 times volume of normal saline using high-throughput tissue grinder (SCIENTZ-48, Ningbo Xingzhi Biotechnology Co. Ltd., China). The tissue homogenate (0.2 mL) was mixed with 1.0 mL of ethyl acetate to precipitate proteins, then the mixture was vortexed vigorously for 2 min and centrifuged at 10 000 rpm for 10 min. The upper organic layer was collected and evaporated at 40 °C under nitrogen. The residue was reconstituted in 200 μL of acetonitrile, and 5 μL was injected for HPLC analysis. All parameters are calculated by using Phenix WinNonlin software, version 6.1.

### *In vivo* antitumor activity

*In vivo* anticancer activity was evaluated using H22 tumor-bearing mice. Method of establishing animal models was the same as described in “*In vivo* biodistribution study”. The mice were randomly divided into five groups (10 mice per group), among which three groups were intravenously administrated with CUR-NSps (2.5, 5.0 or 10.0 mg/kg respectively), one group was intravenously administrated with CUR injections at the dose of 10.0 mg/kg, and another group was intravenously administrated with 0.2 mL normal saline as a control. All the mice were dosed every other day. During the whole process of experimentation, the body weight and the volume of tumors were monitored every day. The mice were sacrificed after 6 days of treatment. The tumors were harvested and weighted. The *in vivo* tumor inhibition ratio (TIR %) was calculated as follows:

(4) $$\mathrm{TIR}\%=\left(1-W_{t}/W_{n}\right)\times 100\%$$

where, W_n_ is the average tumor weight of negative control group and W_t_ is the average tumor weight of the tested group.

### Statistical analysis

The statistical analysis among the experimental groups was performed using the independent-samples *t*-test and IBM SPSS Statistics software, version 19 (IBM Co., Armonk, NY). *p < 0.05 or less was considered statistically significant.

## Results and discussion

### Preparation of CUR-NSps

**Figure 1.**
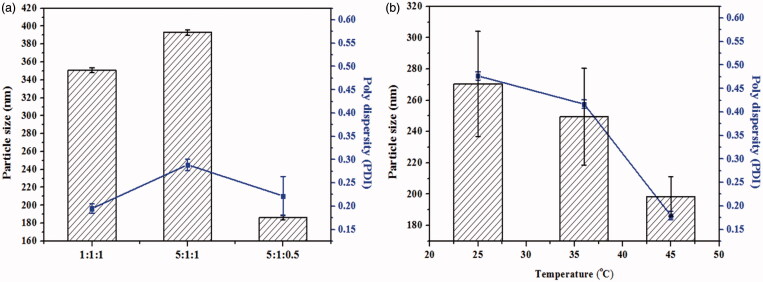
Formulation screening and technique process optimization of CUR-NSps. (a) Particle size and PDI of CUR-NSps with different ratio of drug to stabilizer (w/w/w); (b) particle size and PDI of CUR-NSps prepared at different temperature. All data represent the mean ± SD (*n* = 3).

It is well-known that the stabilizer plays a key role in the successful preparation of NSps. In our trial test, mPEG2000-PCL2000, D-α-tocopherol polyethylene glycol 1000 succinate (TPGS), mPEG2000-PCL2000 combined with SPC, mPEG2000-PCL2000 combined with BSA, mPEG2000-DSPE, and SPC were first tried in our trial test (drug:stabilizer = 1:5, weight ratio). However, none of these stabilizers alone could achieve the CUR NSps with good storage stability more than 3 days, some even precipitated immediately after preparation. However, CUR NSps prepared using mPEG2000-DSPE and SPC as combined stabilizers had the best storage stability of aqueous NSps. To increase the drug payload while maintaining the small size and stability, different CUR/mPEG2000-DSPE/SPC ratios (1:1:1, 5:1:1, 5:1:0.5, w/w/w) were investigated for the preparation of CUR-NSps. As seen in Figure 1(a), when the drug-stabilizer ratio was increased from 1:1:1 to 5:1:1, the particle size increased from 350.8 nm to 392.9 nm. This is because when the amount of lipophilic drug increased, the resultant nanosuspensions have to be enlarged in size to accommodate the relative remnant drugs with limited stabilizer. However, when the mass ratio of SPC decreased from 5:1:1 to 5:1:0.5, the particle size decreased significantly from 392.9 nm to 186.3 nm. The presence of small amount of SPC in the formulation may reduce the steric repulsion produced by PEG chains and make CUR-NSps more stable. As the ratio of SPC increased, the excess SPC might participate in the self-organization of the large size, vesicle-like structures (Khan et al., 2013). Besides, the actual drug payload of CUR-NSps for the three drug-stabilizer ratios (1:1:1, 5:1:1, 5:1:0.5) was 18.97 ± 0.04%, 34.26% ± 0.25, and 67.07 ± 1.03%, respectively (w/w, actual determination). As a result, a CUR/mPEG2000-DSPE/SPC ratio of 5:1:0.5 with the smallest particle size and highest drug payload was chosen for the subsequent preparation of CUR NSps.

Different sonication temperatures (25, 37, and 45 °C) could also affect the particle size of the resultant CUR-NSps. As demonstrated in Figure 1(b), the size of the obtained CUR-NSps reduced as the sonication temperature increased, and the smallest size was achieved when the temperature was up to 45 °C. This might be because this temperature helps guarantee the relative rapid nucleation and the relative slow growth of nucleus, which results in small particles (Rabinow, 2004). Therefore, 45 °C was chosen as the final preparation temperature.

### Particle size, zeta potential, and morphology

**Figure 2.**
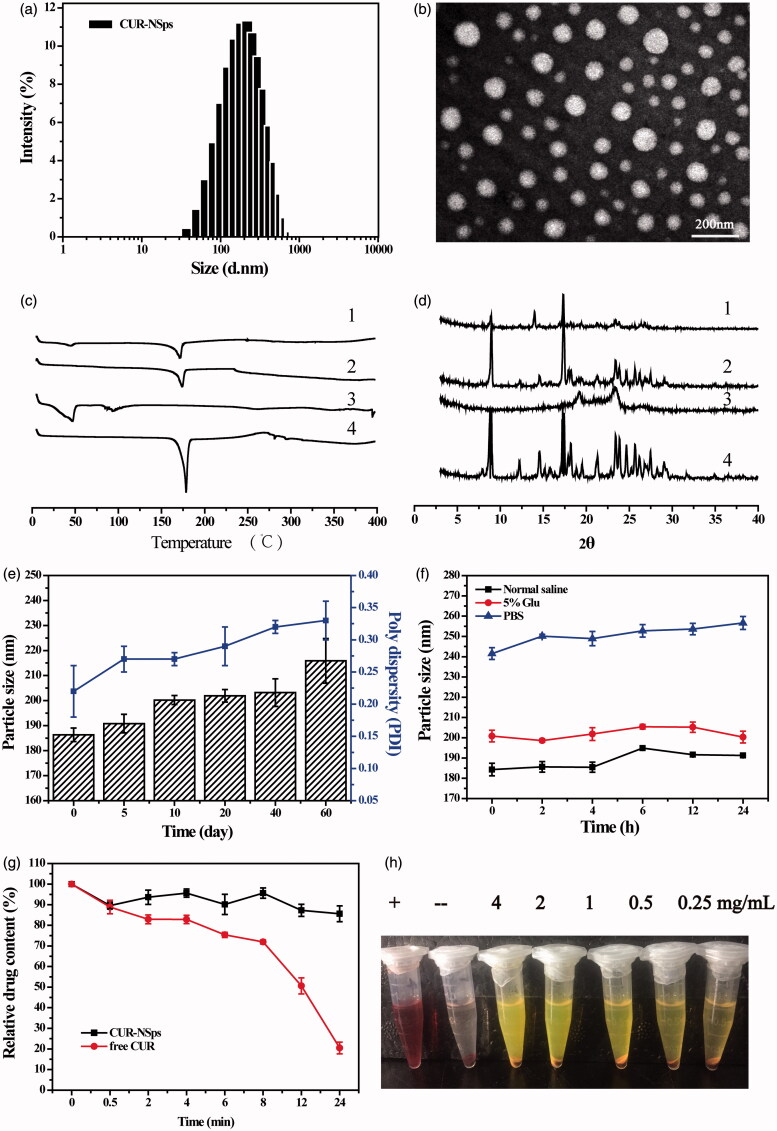
Physical characterization and the stability of CURs-NSps. (a) The particle size of CUR-NSps measured by DLS. (b) TEM micrograph of CUR-NSps. The scale bar is 200 nm. (c) DSC thermograms for 1: CUR-NSps; 2: physical mixture of CUR and excipients; 3: blank excipients; 4: CUR bulk powder. (d) The XRD patterns of 1: CUR-NSps; 2: physical mixture of CUR and excipients; 3: blank excipients; 4: CUR bulk powder. (e) Particle size change of CUR-NSps during the storage at 4 °C for 60 days. (f) Particle size changes of CUR-NSps in normal saline, 5% Glu and PBS at 37 °C. (g) Relative drug content of CUR-NSps and free CUR incubated with plasma at 37 °C. (h) The hemolysis assay of CUR-NSps at different concentrations. From left to right: positive control, negative control, 4 mg/mL, 2 mg/mL, 1 mg/mL, 0.5 mg/mL, 0.25 mg/mL of CUR-NSps.

In this study, antisolvent precipitation-ultrasonication method was proved to be a simple but quite effective approach to prepare CUR-NSps. The average diameter of the resultant CUR-NSps was 186.33 ± 2.73 nm (Figure 2a) with a PDI value of 0.22. The zeta potential was −19.00 ± 1.31 mV. Transmission electron microscope (TEM) observation (Figure 2b) revealed that the CUR-NSps were regular and spherical in shape. TEM micrographs demonstrated that the CUR-NSps were smaller than those that were observed by DLS examination because the drying process resulted in particle shrinkage.

### Differential scanning calorimetry analysis

DSC thermogram of CUR bulk powder, blank excipients, CUR-NSps, and physical mixture of CUR bulk powder and excipients are shown in Figure 2(c). Thermogram of CUR showed a sharp endothermic peak at 178.2 °C, which corresponds to the melting point of CUR. No melting peak was detected in blank excipients. The melting endothermic peak and melting temperature of CUR-NSps were almost identical to the drug bulk powder, suggesting precipitation-ultrasonication method did not change the crystalline form of CUR during the preparation of CUR-NSps.

### X-ray diffraction (XRD) measurements

To support the findings in DSC study, analysis of XRD patterns of CUR bulk powder, blank excipients, CUR-NSps, and physical mixture of CUR bulk powder and excipients were performed (Figure 2d). The diffractogram of CUR bulk powder showed sharp and intense peaks of crystallinity. In the lyophilized CUR-NSps, the diffraction pattern was consistent with that of the bulk powders, suggesting that CUR also existed in the crystalline form in the resultant NSps.

### The stability of CUR-NSps

The physical stability of the aqueous CUR-NSps was evaluated at 4 °C for 60 days. During this storage period, the particle size of the samples was consistent with that of the freshly prepared CUR-NSps, and the CUR content was not significantly changed (Figure 2e), indicating that the aqueous CUR-NSps had a shelf-life of at least 2 months at 4 °C. The stability of CUR-NSps was also evaluated at room temperature. The particle size was increased about 50 nm in 10 days (data not shown), indicating that CUR-NSps was relatively more stable at 4 °C than room temperature. This is because the formation of hydrophobic interactions between the nanosuspensions and stabilizer is a negative entropic process. Thus, the higher the temperature, the more thermodynamically unfavorable the system stability becomes. Thus, in the hydrophobic nanosuspension systems, the tendency of aggregation is enhanced at higher temperature (Deng et al., 2010).

The obtained CUR-NSps were quite stable in normal saline and 5% glucose solution (Figure 2f) after incubation at 37 °C for 24 h despite a relatively larger size than that in deionized water. The particle size of CUR-NSps increased approximately 80 nm in PBS. In general, the saline environment (i.e. ionic strength, pH) could often affect the surface charge and zeta potential of the NSps, and sometimes reduce the thickness of electronic double layer or the hydration layer of the NSps while consequently induce the aggregation of the NSps (Owen et al., 2009). PBS contains different saline ions such as: Na^+^, K^+^, Cl^−^, HPO_4_^−^, H_2_PO_4_^−^. Since CUR-NSps are stable in 0.9% NaCl, Cl^−^, and Na^+^ was proved to have little effect on the stability of CUR-NSps. It should be K^+^, HPO_4_^−^, H_2_PO_4_^−^ that caused size enlargement of CUR-NSps in PBS, which has been proved by our subsequent stability test of CUR-NSps in KH_2_PO_4_ and K_2_HPO_4_ solutions. Therefore, the dispersion medium for CUR-NSps was adjusted to an isotonic solution of 0.9% sodium chloride for i.v. administration in the subsequent experiment.

The drug content changes of CUR-NSps and free CUR incubated with plasma were also determined at 37 °C for 24 hours. As demonstrated in the Figure 2(g), there was still 85.64% of drug retained in CUR-NSps after 24 h incubation with plasma. However, only 20.48% of drug was left for free CUR. Therefore, the obtained CUR-NSps could well protect the encapsulated CUR from rapid metabolism by plasma enzymes and so on. The results of hemolysis assay (Figure 2h) showed that CUR-NSps demonstrated lower hemolysis at all the tested concentrations (<5%), indicating that CUR-NSps was safe and met the demand of intravenous injection.

### *In vitro* cytotoxicity assay

**Figure 3.**
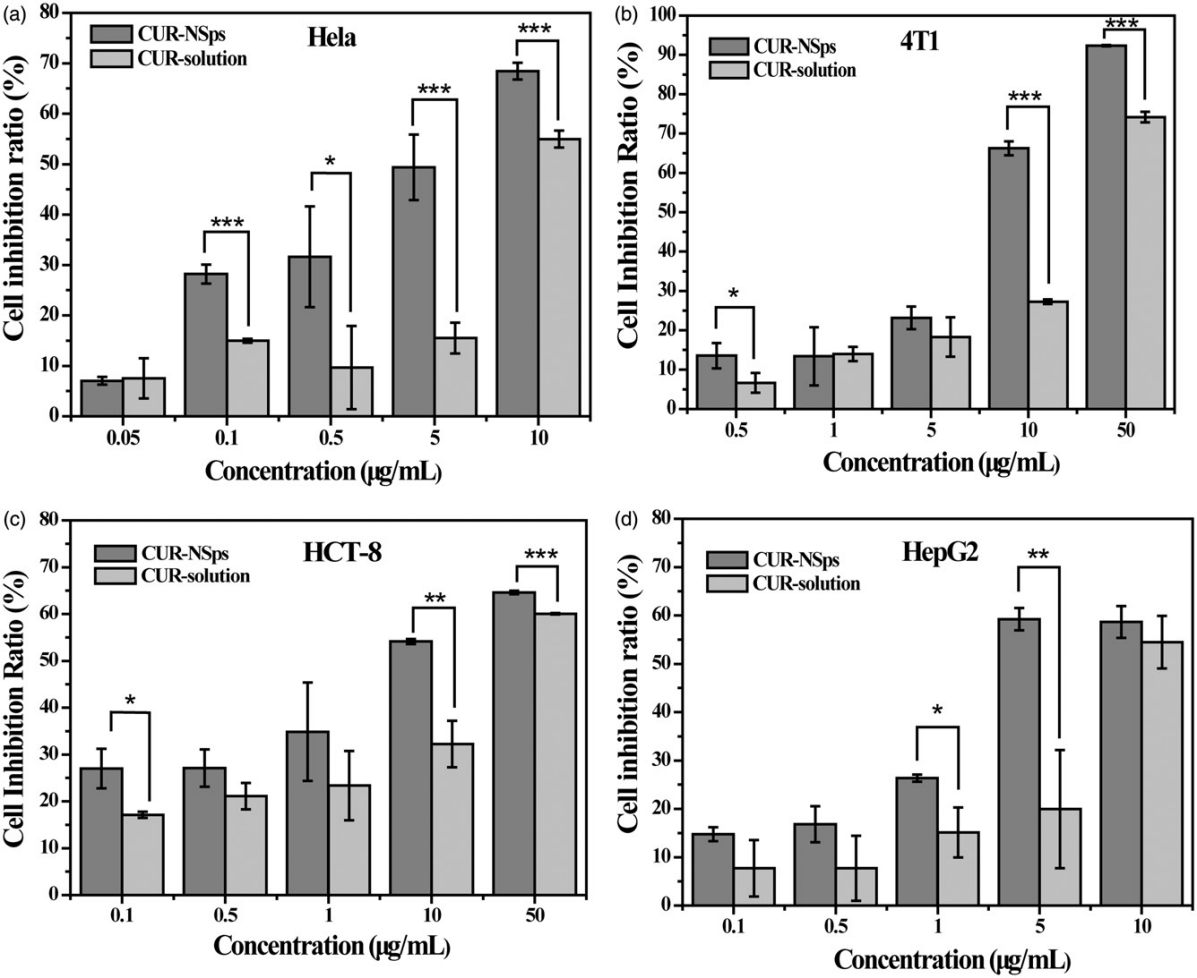
The *in vitro* antiproliferative activity of CUR-NSps and free CUR solution. (a) Cytotoxicity of CUR-NSps and CUR solution against Hela cells. (b) Cytotoxicity of CUR-NSps and CUR solution against 4T1 cells. (c) Cytotoxicity of CUR-NSps and CUR solution against HCT-8 cells. (d) Cytotoxicity of CUR-NSps and CUR solution against HepG2 cells. All the samples were incubated with cells for 48 h by MTT assay. The results are presented as the mean ± SD, *n* = 6. *p < 0.05, **p < 0.01, ***p < 0.001.

An MTT assay was used to assess the *in vitro* cytotoxicity of the CUR-NSps compared to the free CUR solution against Hela, 4T1, HepG2, and HCT-8 cell lines. As shown in Figure 3(a–d), both CUR-NSps and CUR solution inhibited the growth of these tumor cells in a dose-dependent manner. CUR-NSps exhibited higher cytotoxicity than CUR solution at nearly each concentration of drug. Data from Table 1 indicated, according to the IC_50_ values, that CUR-NSps were 2.24, 1.64, 3.49, and 1.70 times more effective than CUR solution against Hela, 4T1, HCT-8, and HepG2, respectively.

**Table 1.** IC_50_ values (μg/mL) of CUR-NSps and free CUR solution against four tumor cell lines after incubation for 48 h.

IC_50_ (μg/mL)	CUR-NSps	CUR-solution
Hela	2.871 ± 1.286*	9.300 ± 1.519
4T1	7.905 ± 1.701**	20.860 ± 4.45
HCT-8	6.911 ± 3.41	31.04 ± 16.16
HepG2	4.296 ± 1.058	11.58 ± 4.926

The results are presented as mean ± SD, *n* = 6.

* *p* < 0.05 versus CUR solution.

** *p* < 0.01 versus CUR solution.

The enhanced *in vitro* cytotoxicity of CUR-NSps may be attributed to nanoparticles that were attached nonspecifically to the cell membrane and then were internalized in the tumor cells via endocytosis (Dong et al., 2016; El-Khoury & Patra, 2016). Additionally, the SPC in the CUR-NSps may enhance their adhesion and uptake by tumor cells. These factors have been previously reported (Lou et al., 2009; Gao et al., 2011).

### Pharmacokinetic analysis

The plasma concentration of CUR was calculated by HPLC method. The limit of quantification (LOQ) for CUR in plasma sample was 0.02 μg/mL. The standard curves with CUR concentrations ranging from 0.02 to 5.0 μg/mL exhibited good linearity for all measured samples (*R*^2^ = 0.999). The extraction recoveries of CUR at the concentrations of 0.05, 0.50, and 5.0 μg/mL from plasma samples were all more than 85%.

**Figure 4.**
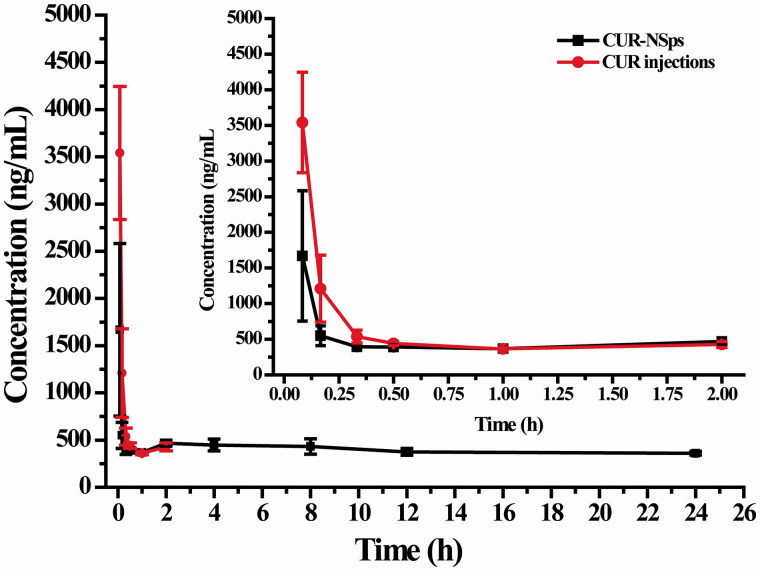
The mean plasma concentration of CUR after intravenous administration of CUR-NSps and CUR injections at a single dose of 10 mg/kg body weight (mean ± SD, *n* = 10).

As compared to free CUR injections, CUR-NSps with a suitable size (<200 nm) usually show a longer retention time in the bloodstream (Barreto et al., 2011). To confirm this hypothesis, the pharmacokinetic study was undertaken by i.v. injection of CUR-NSps and CUR injections. The plasma drug concentration versus time profiles of the CUR obtained after i.v. administration of the CUR-NSps and CUR injections are shown in Figure 4. We observed that the plasma concentration of CUR in the injections was undetectable after 240 min, but the group treated with CUR-NSps exhibited a higher plasma drug concentrations more than 450 ng/mL at this time-point and maintained at similar drug level approximately till 1440 min. Within 30 min after the intravenous injection, plasma drug concentration of the CUR-NSps was less than that of the injection group. This may be due to the quicker diversion of CUR-NSps from blood to organs, especially reticuloendothelial system (RES) such as liver and spleen once entering the circulation system, which has been extensively reported (Gao et al., 2010). The pharmacokinetic parameters presented in Table 2 showed significant differences between the CUR-NSps and the injections groups. The elimination half-life value of CUR-NSps (*t*_1/2_, 65.07 ± 30.88 h) was approximately 35.95-fold that of CUR injections (1.81 ± 1.56 h, *p* < 0.01). The AUC_0–24_ value of CUR-NSps was 4.50 times that of the injections group (7672.04 h × ng/mL versus 1704.69 h × ng/mL, *p* < 0.05). The mean residence time (MRT, 9.45 ± 3.28 h) for the CUR-NSps was significantly longer (18.90-fold) than that of CUR solution (*p* < 0.01). The longer blood retention time of CUR-NSps provides the possibility of enhanced drug accumulation in the tumor tissues.

**Table 2.** Pharmacokinetic parameters after i.v. administration of CUR-NSps and CUR injections.

	CUR-NSps	CUR injections
*t*_1/2_ (h)	65.07 ± 30.88**	1.81 ± 1.56
*C*_max_ (ng/mL)	1467.08 ± 215.06**	3696.13 ± 311.57
AUC_last_ (h*ng/mL)	7672.04 ± 2594.79*	1704.69 ± 122.52
Cl (mL/h/kg)	209.42 ± 164.61**	3822.45 ± 976.68
MRT (h)	9.45 ± 3.28**	0.50 ± 0.04

The results are presented as mean ± SD, *n* = 10.

* *p* < 0.05 versus CUR injections.

** *p* < 0.01 versus CUR injections.

The possible reason was that the CUR-NSps, which was stabilized by hydrophilic polymer, might have a reduced opsonization and a longer circulation time, which was consistent with the reported results (Kim et al., 2001; Hu et al., 2013,2014). High mobility of linear poly (ethylene glycol) chains in mPEG2000-DSPE might repel the approach of proteins such as opsonins to the surfaces of CUR-NSps, thus reduced the clearance of CUR-NSps from the blood and prolonged the MRT of CUR in blood (Torchilin, 1998). Meanwhile, only released drug from CUR-NSps could be metabolized by various enzymes in blood. On the contrary, the drug in CUR injection was directly and quickly metabolized (Prasad et al., 2014) once entering into blood and therefore disappeared quickly from blood and undetectable by HPLC analysis.

### *In vivo* biodistribution study

**Figure 5.**
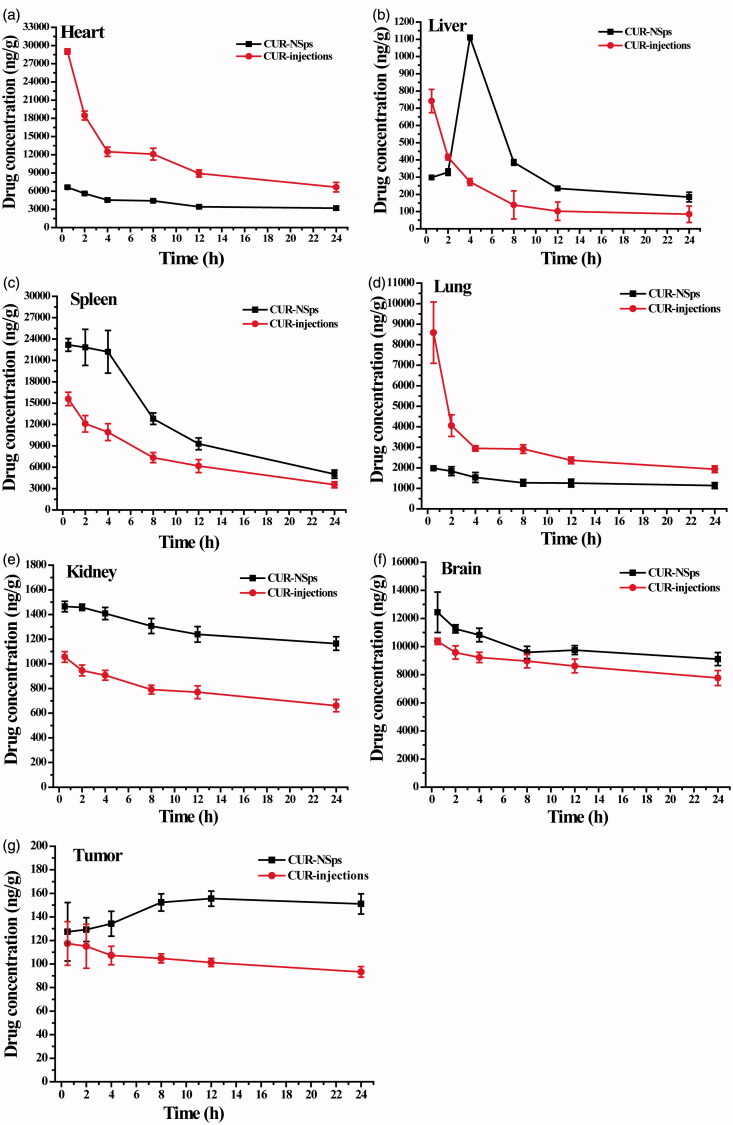
Biodistribution of CUR after intravenous administration of CUR-NSps and CUR injections in H22 tumor-bearing mice at the same dose of 8 mg/kg body weight (mean ± SD, *n* = 10).

Figure 5 and Table 3 demonstrated the drug concentration and accumulation in different tissues after intravenous administration of CUR-NSps and injections at a dose of 8 mg/kg. The CUR-NSps exhibited a higher drug level in liver, spleen, kidney, brain, and tumor.

**Table 3.** AUC_0–24 h_, MRT and *C*_max_ of the biodistribution of CUR-NSps and CUR injections.

	AUC_0–24 h_/h·μg·mL^−1^		MRT/h		*C*_max_/μg·mL^−1^	
	CUR-NSps	CUR injections	CUR-NSps	CUR injections	CUR-NSps	CUR injections
Heart	134.28 ± 13.67*	203.25 ± 27.04	11.25 ± 0.26	9.96 ± 1.03	6.85 ± 0.14	19.00 ± 1.35
Liver	7.33 ± 0.44***	3.52 ± 0.24	11.15 ± 0.21*	8.44 ± 1.13	0.40 ± 0.02	0.41 ± 0.06
Spleen	419.70 ± 27.25**	319.03 ± 13.83	13.55 ± 0.53	14.19 ± 0.61	25.30 ± 3.36	20.30 ± 1.23
Lung	84.87 ± 9.95	96.28 ± 29.29	12.27 ± 1.24	9.11 ± 2.90	6.03 ± 1.87	17.94 ± 3.84
Kidney	37.61 ± 1.85**	27.22 ± 1.57	12.13 ± 0.32	11.99 ± 0.90	1.72 ± 0.07*	1.41 ± 0.16
Brain	301.17 ± 5.31*	263.37 ± 19.57	11.91 ± 0.47	11.78 ± 0.47	14.07 ± 0.52	13.69 ± 0.14
Tumor	3.56 ± 0.04**	2.70 ± 0.27	12.21 ± 0.20	11.78 ± 0.47	0.16 ± 0.01***	0.13 ± 0.01

The results are presented as mean ± SD, *n* = 10.

*p < 0.05 vs CUR injections.

**p < 0.01 vs CUR injections.

***p < 0.001 vs CUR injections.

Take AUC_0–24 h_ as an example, administration of CUR-NSps led to 2.08, 1.31, 1.38, 1.14, and 1.32 times AUC_0–24 h_ as CUR injections in drug biodistribution in liver, spleen, kidney, brain, and tumor, respectively. The higher drug distribution in tumor for CUR-NSps group was mainly due to the enhanced permeability and retention (EPR) effect (Jain, 2005; Yuan et al., 2015). Remarkably, the drug concentration in the tumors of CUR injection group decreased over time; however, for CUR-NSps group, the drug distribution in tumor showed a slow increase in the first 8 h and then maintained at a much higher concentration than CUR injections group till 24 h (151.05 ng/g versus 93.31 ng/g, *p* < 0.001). This may be because the NSps accumulated in liver, spleen, and lung could gradually release the encapsulated drug (Chiannilkulchai et al., 1990; Soma et al., 2000) and then partially transferred to tumor through blood circulation.

The levels of CUR-NSps accumulated in spleen tissue might be closely related to phagocytic cell uptake in the RES system (Moghimi et al., 2001). Kim et al. reported that when intravenous administration of water-soluble albumin bound-CUR-NSps (about 135 nm in size with a zeta potential of −23.4 mV), a great quantity of CUR distributed in the liver instead of spleen (Kim et al., 2011). However, CUR-loaded PLGA NSps (about 163 nm in size with a zeta potential of −12.5 mV) exhibited enhanced biodistribution in spleen, much higher than that in the liver (Tsai et al., 2011). These results together with our findings implied that the property of pharmaceutical adjuvants in the formulation might influence the *in vivo* biodistribution.

Figure 5(f) showed that both CUR-NSps and injections group had obvious drug distribution in brain, which demonstrated that both CUR-NSps and injections group could pass through the blood brain barrier (BBB) into brain tissue. But CUR-NSps demonstrated a higher CUR concentration in brain at all time-points, which might be the result of transport of the CUR-NSps through the blood-brain barrier by endocytosis and sustained diffusion release of CUR from nanosuspensions (Minagawa et al., 1996; Tsai et al., 2011; Chen et al., 2016).

### *In vivo* antitumor efficacy

**Figure 6.**
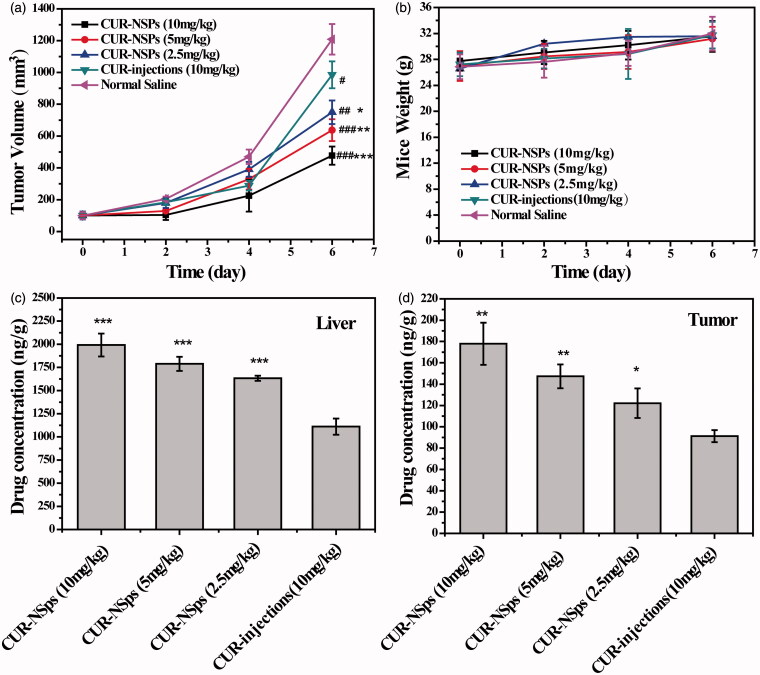
The *in vivo* antitumor efficacy of CUR-NSps and CUR injections against H22 tumor-bearing mice after intravenous administration. (a) The growth of tumor volume over time. #*p* < 0.05 versus normal saline, ##*p *< 0.01 versus normal saline, ###*p *< 0.001 versus normal saline. *p < 0.05 versus CUR injections, **p < 0.01 versus CUR injections, ***p < 0.001 versus CUR injections. (b) The body weight change of mice with time. (c) The drug concentration of CUR-NSps and CUR injections in liver after multiple intravenous administration. ***p < 0.001 versus CUR injections. (d) The drug concentration of CUR-NSps and CUR injections in tumor after multiple intravenous administration. *p < 0.05 versus CUR injections, **p < 0.01 versus CUR injections. All data represent the mean ± SD, *n* = 10.

To determine the therapeutic advantages of CUR-NSps, the *in vivo* antitumor efficacy of CUR-NSps and injections were investigated using H22 tumor-bearing mice. Figure 6(a) showed the variation of tumor volume throughout the whole experiment. The tumor volume in the saline control group increased rapidly and reached a 12-fold increase in volume at the end of the trial. The three doses of CUR-NSps (10 mg/kg, 5 mg/kg, and 2.5 mg/kg) demonstrated limited tumor growth (4.77, 6.37, and 7.50-fold, respectively) in comparison to 10 mg/kg of CUR injections (9.85-fold). This suggests that CUR-NSps are much more potent than CUR injections. The body weight change of the mice in each group was also measured to evaluate the toxicity of the tested drugs (Figure 6b). All the mice in the tested groups displayed continuous weight increase with time, indicating good tolerance and no significant harm.

Table 4 listed the tumor inhibition rate (TIR) against H22 tumor for all the groups. At the same dose of 10 mg/kg, CUR-NSps demonstrated superior antitumor efficacy over CUR injections (70.34% versus 40.03%, *p* < 0.01). Even at half dose (5 mg/mL), CUR-NSps could achieve significantly higher TIR than CUR injections (55.98% versus 40.03%, *p* < 0.05). When the dose was further reduced to 1/4 (2.5 mg/mL), CUR-NSps still demonstrated higher TIR than CUR injections (53.21% versus 40.03%, *p* > 0.05) though no significant difference was observed. This result was consistent with that of tumor volume increase.

**Table 4.** The *in vivo* antitumor effects of different groups of CUR-NSps against H22 tumors in mice.

Sample	Dose (mg/kg)	Tumor weight (g)	Inhibition rate (%)
CUR-NSps	10	0.416 ± 0.060###,**	70.34
CUR-NSps	5	0.618 ± 0.031##,*	55.98
CUR-NSps	2.5	0.657 ± 0.050##	53.21
CUR injection	10	0.842 ± 0.111#	40.03
Normal saline	–	1.404 ± 0.265	–

The tumor weight results are presented as the mean ± SD, *n* = 10.

# *p* < 0.05 versus normal saline.

## *p* < 0.01 versus normal saline.

###*p* < 0.001 versus normal saline.

* *p* < 0.05 versus CUR injections.

** *p* < 0.01 versus CUR injections.

It was quite clear that when formulated into NSps as in this study, CUR would greatly improve its antitumor efficacy in comparison with CUR solution. The reasons can be explained as follows. First, the long-circulated and sustained drug release of CUR-NSps, as proved by the pharmacokinetic analysis, could provide much more active CUR in blood and tumor to exert the antitumor effect. Second, more CUR-NSps could accumulate in tumor than CUR injections due to their nanometered particle size and the resultant EPR effect. This has been proved by the above biodistribution data after single-dose. Beside, CUR-NSps demonstrated much more potent antitumor cytotoxicity than free CUR. At the end of the *in vivo* antitumor trial, the livers and tumors from different groups were excised and analyzed using HPLC for determining the actual concentration of CUR after multiple-dose. Figure 6(d) displayed that in comparison with single-dose, multiple-dose of CUR-NSps led to more drug concentration in tumor than CUR injections (1.62-fold versus 1.95-fold). This might be another reason for the significantly enhanced antitumor efficacy.

## Conclusions

In this study, CUR-NSps with a high drug payload of 67.07% were successfully prepared by precipitation-ultrasonication method using mPEG2000-DSPE and SPC. The obtained CUR-NSps presented a sphere-like shape by TEM with a mean particle size of 186.33 nm. CUR-NSps presented a much higher cytotoxicity than free CUR solution against Hela, 4T1, HCT-8, and HepG2 cell lines *in vitro*. The results of pharmacokinetic studies revealed a significantly greater AUC_0–24_ and prolonged MRT of CUR-NSps compared to CUR injections after i.v. administration. Tissue biodistribution study in mice indicated higher CUR levels in liver, kidney, brain, and tumor for CUR-NSps compared with CUR injections. The *in vivo* antitumor studies in H22 tumor-bearing mice demonstrated that CUR-NSps could achieve much better therapeutic efficacy than CUR injection at the same dose (10 mg/kg). Therefore, CUR-NSps might be a good choice for intravenous administration of poorly soluble CUR.
